# Historical analysis of the directors of university institutes of pharmacology in Germany and Austria in the period 1918–1963

**DOI:** 10.1007/s00210-025-04342-1

**Published:** 2025-06-27

**Authors:** Hannes Schneider, Christine Wolters, Roland Seifert

**Affiliations:** 1 Institute of Pharmacology, Hannover Medical School, Carl-Neuberg-Straße 1, 30625 Hannover, Germany; 2 Institute for Ethics, History and Philosophy of Medicine, Hannover Medical School, Carl-Neuberg-Straße 1, 30625 Hannover, Germany

**Keywords:** History of pharmacology, Nazi era, Pharmacological ordinaries, Data analysis

## Abstract

In the National Socialism, pharmacology was very important. The First World War had shown the significance of chemical warfare agents, medicines were needed to treat malaria and typhoid fever, and soldiers had to be fit for fighting as long as possible. But there are large gaps in the history of German-speaking pharmacology around the period of National Socialism until today. Using the example of the directors of university pharmacological institutes in Germany and Austria between 1918 and 1963, this work aims to show how pharmacologists, like doctors in other disciplines, got expelled by the Nazis or became a part of the political racist and extreme system of the National Socialism. For this purpose, detailed information was collected for each institute director under consideration through research in the federal, state, and university archives, the archives of the German Society for Experimental and Clinical Pharmacology and Toxicology (Deutsche Gesellschaft für experimentelle und klinische Pharmakologie und Toxikologie e.V., DGPT), and the specialist literature. The results were presented in graphical form. This study finds that almost two-thirds of institute directors (63%) were members of the National Socialist German Workers’ Party (Nationalsozialistische Deutsche Arbeiterpartei, NSDAP) and/or other National Socialist organizations between 1933 and 1945. About half (51%) of the consulting pharmacologists of the German army worked as institute directors at universities. In addition, around 40% of the institute directors conducted research directly for the German military. By looking at the reasons for expulsions, the political and racial persecution, the influence of the National Socialists on the German pharmacological community can be revealed. This work also shows the expulsion of Jewish professors from 1933 onwards and the failed attempts at denazification by the Allies after 1945. The study is the first to consolidate existing data and previously unconsidered archive material into an overall picture of the development of university pharmacological institutes during the Nazi era. The study shows deep involvement of the German pharmacological community in the National Socialist system.

## Introduction

“Inevitably and fatefully, the age of chemistry has also influenced the methods of national defense and warfare. In the numerous tasks associated with this, […] pharmacological work is involved in a fundamental and trendsetting way.” [Transl. by the author; “Zwangsläufig und schicksalsmäßig hat das Zeitalter der Chemie auch die Methoden der Landesverteidigung und Kriegsführung beeinflußt. Bei den zahlreichen damit verbundenen Aufgaben, […] ist die pharmakologische Arbeit grundlegend und richtungweisend beteiligt.”] (Flury 1938).

With these words, Ferdinand Flury opened the congress of the German Pharmacological Society in Berlin in 1938. They show the extent to which German pharmacology was still under the influence of the First World War and how it understood its task at this time. Two years earlier, Walther Straub had already used similar words in his opening speech in Munich and offered the support of the Pharmacological Society to the National Socialists:

“We have always been interested in the toxicology of the air, the study of gas warfare poisons and, in particular, the discovery of antidotes, cures and treatment for the effects of poisoning, which has moved into our sphere of interest from outside. The happy rebirth of our armed forces will give us the opportunity to deal with these problems scientifically on a larger scale. We offer our services.” [Transl. by the author; “Die von außen her in unseren Interessenkreis gerückte Toxikologie der Luft, das Studium der Gaskampfgifte und besonders das Auffinden von Gegengiften, Heilmitteln und Behandlung von Vergiftungsfolgen, hat uns stets interessiert. Die glückliche Wiedergeburt unserer Wehrmacht wird uns Gelegenheit geben, in erweitertem Umfang diese Probleme wissenschaftlich zu behandeln. Wir bieten unsere Dienste an.”] (Straub 1936).

This quote from Walther Straub shows that he welcomed National Socialism, as did many other scientists at the time. Straub, who was 62 years old by then, was at the peak of his career as a university lecturer and scientist. He did not become a member of a Nazi organization, so that his attitude was not reflected in the files (BayHStA, MK 18006; BArch R 4901/13,278; UAM E-II-3283). Nonetheless, membership of Nazi organizations is taken in this work as an important indication of a National Socialist mentality to provide an initial overview of pharmacologists under National Socialism and therefore set the scene for further research.

The pharmacological research resulting from this mindset, on chemical weapons such as Tabun and Sarin, as well as on malaria medication and pep pills for soldiers such as Pervitin, has already been examined in other works, such as those by Wolfgang U. Eckart und Alexander Neumann (2006) or Florian Schmaltz (2017). After the defeat of Nazi Germany and its occupation by the Allies in 1945, it took several years for many parts of German society to begin to come to terms with their own deeds and actions under National Socialism. In particular, the German medical community refused to take responsibility for medical crimes. It repressed, sometimes concealed or played down its passive and active support for the National Socialism. Officials in the medical industry, clinics, and universities were mostly able to retain their posts with impunity (Frei 2001). Only decades later, individual disciplines began to consciously examine and reflect on their actions, activities and failings during this period (Oehler-Klein and Roelcke 2007; Krischel et al. 2016). One example of this is psychiatry, some of whose representatives committed serious crimes under the Nazi euthanasia program (Schmuhl 2015; Fehlemann et al. 2017). Another example is the Internal Medicine Association (Forsbach and Hofer 2018).

Leading German pharmacologists tried to take the first steps in presenting the history of German pharmacology including the period from 1933 to 1945: Jürgen Lindner and Heinz Lüllmann’s book (1996) provides an initial overview of the professional biographies of pharmacologists at German-speaking university institutes. In a dedicated series of books, Athineos Philippou (2004, 2007, 2011, 2014, 2017, 2021) collected and comments on reports about various pharmacological institutes, as well as biographies and autobiographies of individual pharmacologists. Konrad Löffelholz and Ullrich Trendelenburg (2008) described the fates of Jewish pharmacologists in Germany under the National Socialism in their book. Mispagel and Seifert (2024) expanded this work by describing the influence of this period on Naunyn–Schmiedeberg’s Archives of Pharmacology (NSAP). Steinert and Seifert analysed the recipients of the Schmiedeberg Medal of the DGPT—the highest award for pharmacologists in Germany (Steinert and Seifert 2025). There are also reports by and about individual pharmacologists. Christina Witte (2006) analyzes the career of the Rostock pharmacologist Peter Holtz. Werner Scheler and Peter Oehme (2002) described the career of the Berlin pharmacologist Friedrich Jung. There are also works on the history of individual universities and faculties: Ralf Forsbach (2023) for example, worked on the history of the medical faculty of the University of Cologne and Rainer Möhler (2020) studied the entire Reich University of Strasbourg between 1940 and 1944. Table 1 shows a selection of international papers dealing with the history of pharmacology in Germany under the National Socialism.

**Table 1:** A selection of international papers dealing with the history of pharmacology in Germany under national socialism

Number	Paper
1	Dats LB, von Haugwitz F, Seifert R. Bibliometric development of Naunyn–Schmiedeberg’s Archives of Pharmacology. Naunyn Schmiedebergs Arch Pharmacol. 2023 Jan;396(1):43–61. 10.1007/s00210-022-02307-2. Epub 2022 Oct 25. Erratum in: Naunyn Schmiedebergs Arch Pharmacol. 2023 Jan;396(1):171. PMID: 36 ,280,660; PMCID: PMC9592544
2	Kessel N. Biographie als Disziplinentradition. Von der Idealisierung des Pharmakologen Wolfgang Heubner (1877–1957) [Biography as discipline tradition. The idealization of the pharmacologist Wolfgang Heubner (1877–1957)]. Med Ges Gesch. 2008;27:133–60. German. PMID: 19 ,830,958
3	Prüll CR. Caught between the old and the new–Walther Straub (1874–1944), the question of drug receptors, and the rise of modern pharmacology. Bull Hist Med. 2006 Fall;80(3):465–89. https://doi.org/10.1353/bhm.2006.0111. PMID: 17 ,147,132
4	Mispagel, M., Seifert R, Biographical analysis of 32 pharmacologists persecuted under the Nazi regime: scientific careers between persecution, emigration, and new beginnings. *Naunyn–Schmiedeberg’s Arch Pharmacol.* 2025. 10.1007/s00210-025-04231-7
5	Philippu A, Seifert R. History of pharmacology:2—The Institute of Pharmacology of the University of Strasbourg: genealogy and biographies. Naunyn Schmiedebergs Arch Pharmacol. 2023 Jan;396(1):19–33. 10.1007/s00210-022-02336-x. Epub 2022 Dec 15. PMID: 36 ,520,164
6	Ludwig L, Seifert R. How does pharmacological and toxicological knowledge evolve? A case study on hydrogen cyanide in German pharmacology and toxicology textbooks from 1878 to 2020. Naunyn Schmiedebergs Arch Pharmacol. 2024 Jun 20. 10.1007/s00210-024-03227-z. Epub ahead of print. PMID: 38 ,900,251
7	Barrett JE, Page C, Michel MC. Perspectives of Pharmacology over the Past 100 Years. Handb Exp Pharmacol. 2019;260:3–16. https://doi.org/10.1007/164_2019_334. PMID: 31 ,823,070
8	Prusek K, Labuzek K, Gonciarz M, Okopień B. Farmakolodzy w obozach III Rzeszy–cześć pierwsza [Pharmacologists in the camps In the Third Reich–part one]. Pol Merkur Lekarski. 2013 Oct;35(208):238–41. Polish. PMID: 24 ,340,898
9	Labuzek K, Prusek K, Gonciarz M, Okopień B. Farmakolodzy w obozach III Rzeszy–cześć druga [Pharmacologists in the camps in the Third Reich–part second]. Pol Merkur Lekarski. 2013 Oct;35(208):242–5. Polish. PMID: 24 ,340,899
10	Trendelenburg U. Pharmacology in Germany. Trends Pharmacol Sci. 1998 Jun;19(6):196–8. https://doi.org/10.1016/s0165-6147(98)01195-x. PMID: 9 ,666,706
11	Hattori Y, Seifert R. Reflections on the 150 th anniversary of Naunyn–Schmiedeberg’s Archives of Pharmacology: past, challenges, and future. Naunyn Schmiedebergs Arch Pharmacol. 2023 Jan;396(1):1–3. 10.1007/s00210-022-02321-4. PMID: 36 ,336,742; PMCID: PMC9788996
12	Pohar M, Hansson N. The “Nobel Population” in Pharmacology: Nobel Prize laureates, nominees and nominators 1901–1953 with a focus on B. Naunyn and O. Schmiedeberg. Naunyn Schmiedebergs Arch Pharmacol. 2020 Jul;393(7):1173–1185. 10.1007/s00210-019-01807-y. Epub 2020 Jan 18. PMID: 31 ,953,675
13	Rubin RP. The evolution of the discipline of pharmacology amid an era of global turbulence: the unique contributions of Otto Krayer (1899–1982). J Med Biogr. 2014 Aug;22(3):127–35. https://doi.org/10.1177/0967772014530798. Epub 2014 Jun 6. PMID: 24 ,906,402
14	Löffelholz K. The persecution of pharmacologists in Nazi Germany and Austria. Naunyn Schmiedebergs Arch Pharmacol. 2011 Mar;383(3):217–25. 10.1007/s00210-010-0560-3. Epub 2010 Sep 17. PMID: 20 ,848,274
15	Anderson R. The singular moral compass of Otto Krayer. Mol Interv. 2005 Dec;5(6):324–9. https://doi.org/10.1124/mi.5.6.1. PMID: 16 ,394,245

The works mentioned above focus on individual pharmacologists, institutes or universities, providing in-depth case analyses. Taken together, findings from this line of research still provide a rather incomplete picture of pharmacology in Germany and Austria. Based on previous academic work and further sources from federal archives, state archives, university archives, and the DGPT archives, this publication provides a more comprehensive picture of the pharmacological community in German-speaking countries in the period 1933 to 1945. The focus of this work is on the directors of German-speaking and university-based pharmacological institutes. These individuals represented the pharmacological scientific community very well and played a key role in shaping it on the approach, during and in the aftermath of the Nazi era. To better assess the development during the years of National Socialism, we extend the observation horizon before National Socialism with the interwar period (1918 to 1933) and after with the postwar period and the Adenauer Era (1945 to 1963). Thus covers the period from 1918 to 1963. Personal information on age, denomination and nationality was compiled and evaluated. In addition, the reasons for the appointment and retirement of the professors, their membership in National Socialist associations, military service in the Second World War, as well as scientific projects relevant to the war were examined.

## Materials and methods

### Data collection and data sources

Prior to data collection, it was determined which university institutes existed in Germany and Austria in the period from 1918 to 1963 (Table 2). Many university institutes were founded and closed in this period, mostly because of the many border shifts in Central Europe between 1914 and 1945. This applies in particular to the university institutes in Breslau, Danzig, Königsberg, Posen, Prague, and Strasbourg. For the university institutes, the heads in the period under consideration were researched. This part of the research was essentially based on previous academic work by Lindner and Lüllmann (1996) and Philippou (2004, 2007, 2011, 2014, 2017, 2021). In addition to these, the information on the individual heads of university institutes was taken from the archival records:

- Membership file of the National Socialist German Workers’ Party (NSDAP), internal NSDAP party correspondence, and personnel files of the Reich Ministry of Science, Education, and Public Education from the Federal Archives in Berlin-Lichterfelde
- Curricula vitae and obituaries of members of the DGPT from the DGPT archive in the archive of the Hannover Medical School
- Denazification files and personnel files from the state and national archives of the federal states
- Denazification files and personnel files from the archives of the individual universities

**Table 2:** University Institutes included in the study. The individual institutes are named as they were in the period under review. These designations may differ from those used today

City	University	Institute	Number in the time frame (Fig. 10)
Berlin	Humboldt-Universität zu Berlin	Pharmacological Institute	I
Berlin	Freie Universität Berlin	Pharmacological Institute	II
Berlin	Akademie der Wissenschaften der DDR	Institute for Circulatory Research	III
Bonn	Rheinische Friedrich-Wilhelm-Universität	Pharmacological Institute	IV
Braunschweig	Technische Universität Braunschweig	Institute of Pharmacology and Toxicology	V
Breslau	Schlesische Friedrich-Wilhelm-Universität	Pharmacological Institute	VI
Danzig	Medizinische Akademie Danzig	Pharmacological Institute	VII
Dresden	Medizinische Akademie „Carl Gustav Carus"	Pharmacological Institute	VIII
Düsseldorf	Medizinischen Akademie Düsseldorf	Pharmacological Institute	IX
Erfurt	Medizinische Hochschule Erfurt	Pharmacological Institute	X
Erlangen	Friedrich-Alexander-Universität	Pharmacological Institute	XI
Frankfurt/Main	Johann Wolfgang Goethe-Universität	Pharmacological Institute	XII
Freiburg/Breisgau	Albert-Ludwigs-Universität	Pharmacological Institute	XIII
Gießen	Justus-Liebig-Universität	Pharmacological Institute	XIV
Göttingen	Georg-August-Universität	Pharmacological Institute	XV
Göttingen		Max Planck Society	XVI
Graz	Karl-Franzens-Universität	Pharmacological-Pharmacognostic Institute	XVII
Greifswald	Ernst-Moritz-Arndt-Universität	Pharmacological Institute	XVIII
Halle/Saale	Martin-Luther-Universität	Pharmacological Institute	XIX
Hamburg	Universität Hamburg	Institute of Experimental and Clinical Pharmacology and Toxicology	XX
Hannover	Tierärztliche Hochschule Hannover	Pharmacological Institute	XXI
Heidelberg	Ruprecht-Karls-Universität	Pharmacological Institute	XXII
Homburg/Saar	Universität des Saarlandes	Pharmacological Institute	XXIII
Innsbruck	Leopold-Franzens-Universität	Pharmacological Institute	XXIV
Jena	Friedrich-Schiller-Universität	Pharmacological Institute	XXV
Kiel	Christian-Albrechts-Universität	Pharmacological Institute	XXVI
Köln	Universität zu Köln	Pharmacological Institute	XXVII
Königsberg	Albertus-Universität	Pharmacological Institute	XXVIII
Leipzig	Universität Leipzig	Pharmacological Institute	XXIX
Leipzig	Universität Leipzig	Institute of Veterinary Pharmacology	XXX
Magdeburg	Medizinische Akademie Magdeburg	Institute of Pharmacology and Toxicology	XXXI
Mainz	Johannes-Gutenberg-Universität	Institute of Pharmacology	XXXII
Marburg	Philipps-Universität	Pharmacological Institute	XXXIII
München	Ludwig-Maximilians-Universität	Pharmacological Institute	XXXIV
Münster	Westfälische Wilhelms-Universität	Pharmacological Institute	XXXV
Posen	Reichsuniversität Posen	Pharmacological Institute	XXXVI
Prag	Karl-Ferdinands-Universität	Pharmacological-Pharmacognostic Institute	XXXVII
Rostock	Universität Rostock	Pharmacological Institute	XXXVIII
Straßburg	Universität Straßburg	Pharmacological Institute	XXXIX
Tübingen	Eberhard-Karls-Universität	Pharmacological Institute	XL
Wien	K. K. Universität	Pharmacological Institute	XLI
Würzburg	Julius-Maximilians-Universität	Pharmacological Institute	XLII

During the data research for this work, it became apparent time and again that many data sets are incomplete. Presumably because much of the data has been lost, destroyed or concealed over time. This can be seen, for example, in the NSDAP membership card index of the Federal Archives, in which around 80% of NSDAP memberships can only be verified with the additional help of other holdings of the Federal Archives (Bundesarchiv 2023).

### Reference periods and key variables of interest

The study period is divided into three sections:

- 1918 to 1933, interwar period
- 1933 to 1945, the period under the National Socialism
- 1946 to 1963, postwar period and Adenauer Era

It should be noted that Austria only became part of Germany after it was occupied by German troops in 1938. The period under review from 1933 to 1945 therefore comprises the territory of the German Reich from 1933 to 1937 without Austria and from 1938 to 1945 including Austria.

The following specific aspects were analyzed for each, the Nazi era and the periods before and after. It was examined which nationalities the institute directors had, which denomination they belonged to, how old they were when they were appointed, whether they had served in the military during the Second World War, and whether there were politically and racial reasons for their appointment and retirement. If and since when they were members of the NSDAP or other National Socialist associations was determined by the Membership file of the NSDAP, internal NSDAP party correspondence, personnel files of the Reich Ministry of Science, Education and Public Education and Denazification files.

We also took their age into account. If the institute directors were already very old or dead in 1933, we assumed that they were no members of National Socialist organizations. In the case of younger institute directors, we oriented ourselves on the Allies, who did not initiate denazification proceedings against people born after 1 January 1919 as part of the “youth amnesia” (Benz 2009). The data collected on these characteristics was compared with that of the entire medical profession as researched by Kater (2000).

In order to place the data in a historical context, the change in the number of German-speaking university institutes over time was examined and also the change in the number of institute directors.

## Results

### Overview

**Fig. 1:**
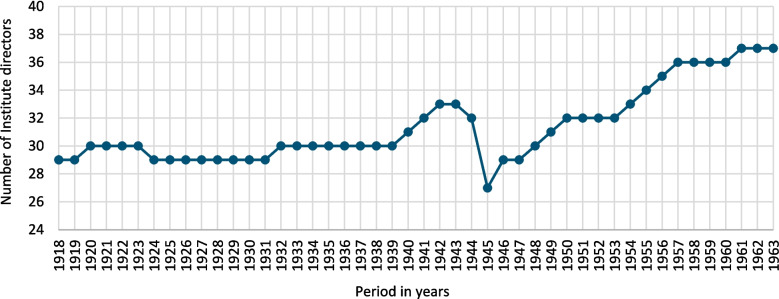
Number of active university pharmacological institutes, at universities, in the individual years from 1918 to 1963, in the territories of Germany and Austria

**Fig. 2:**
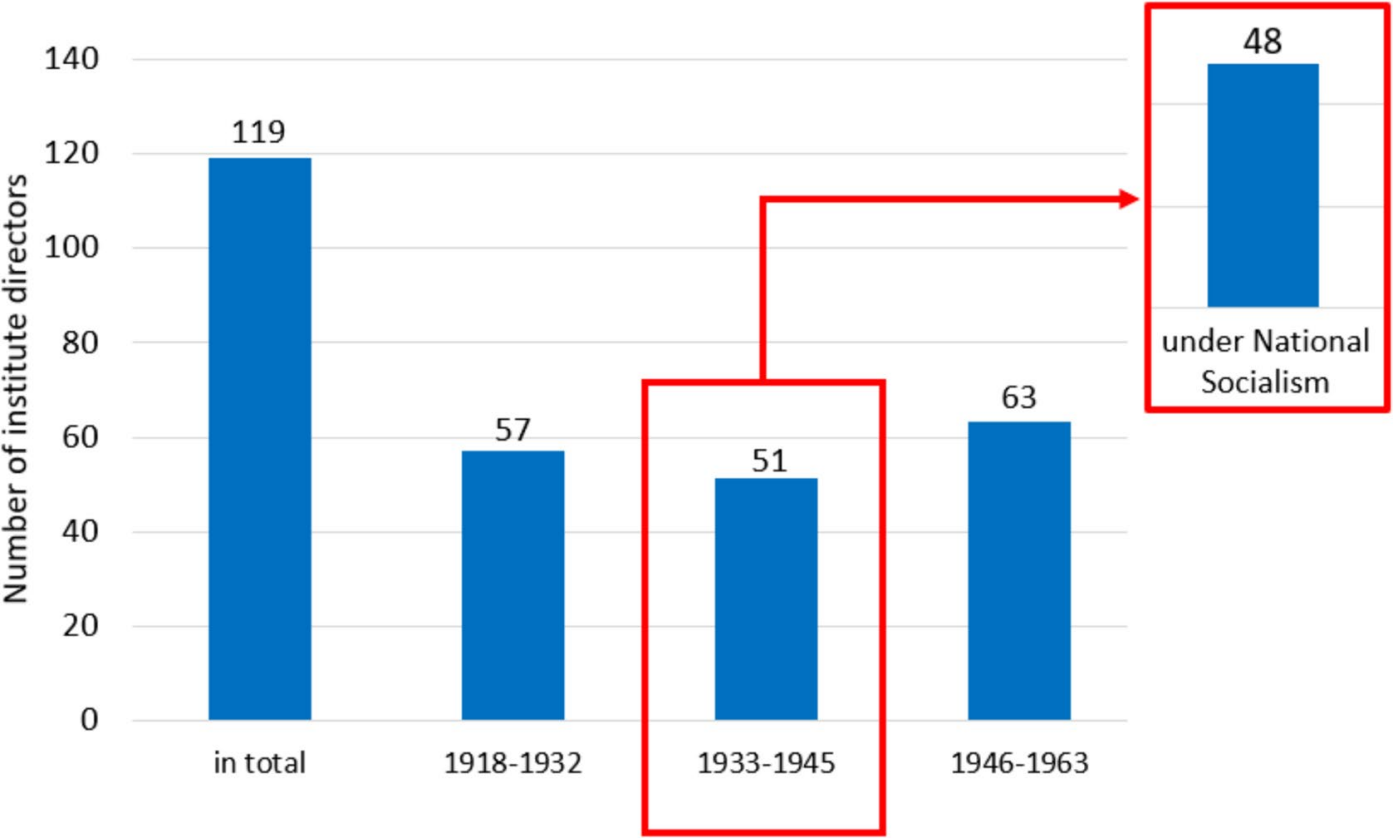
Number of university pharmacological institutes directors at universities in Germany and Austria, first in total from 1918 to 1963, then for the individual periods (1918–1932, 1933–1945, 1946–1963). The difference between the number of directors from 1933 to 1945 and those who worked under National Socialism is emphasized

In total, data was collected from 119 institute directors at 42 university pharmacological institutes. All directors considered as Jewish were removed from office between 1933 and 1938. In long-term view, only one Jewish former chair holder came back to Germany. Of the institute directors, 48 held office under the National Socialism at a total of 33 university institutes (Figs. 1 and 2).

### Institute directors and reasons for change of office from 1918 to 1963

**Fig. 3:**
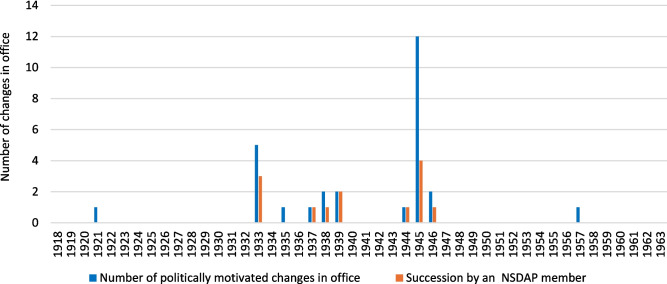
Number of politically and racially motivated office changes for each year from 1918 to 1963 and succession of NSDAP members

**Fig. 4:**
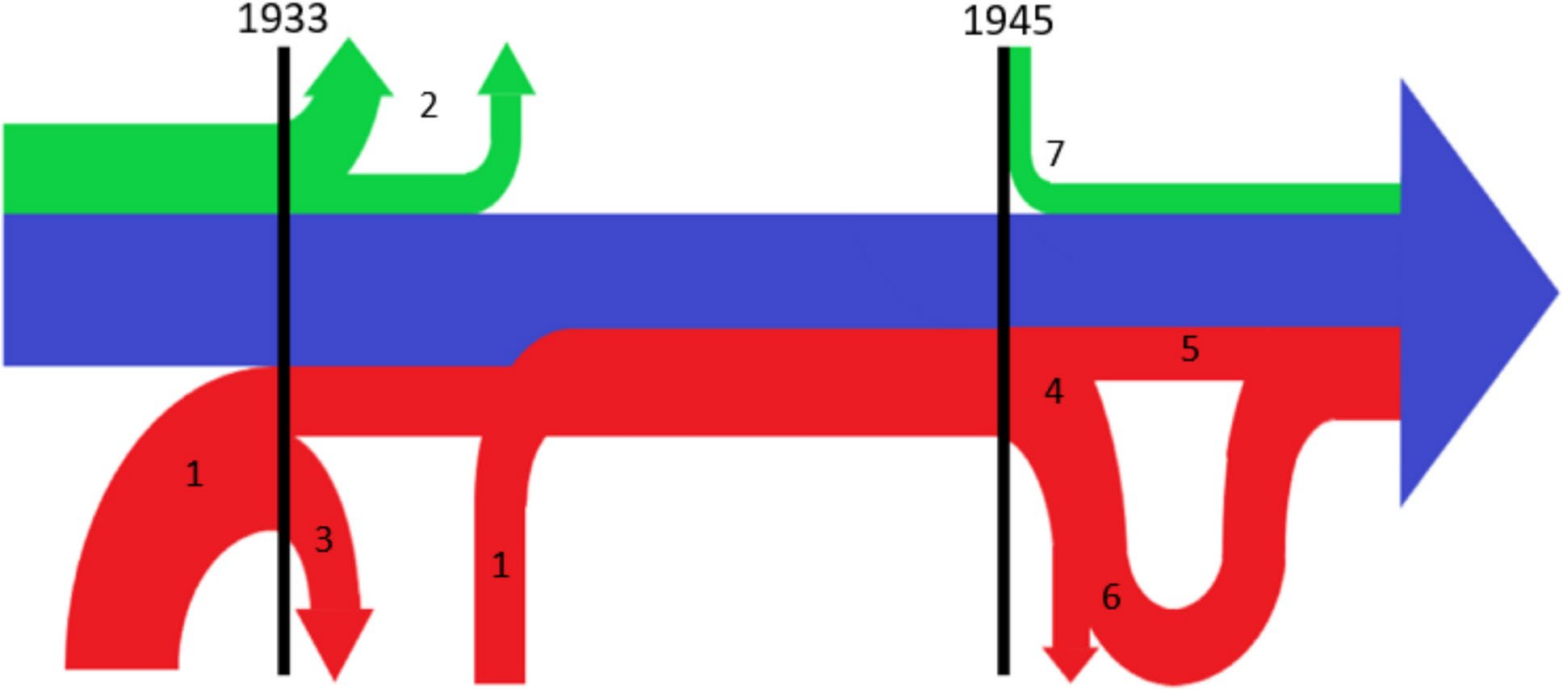
The institute directors over time. Blue line: Institute Directors who worked without long breaks between 1933 and 1945. Green lines: Institute Directors who were Jewish or had Jewish ancestors. Red lines: Institute Directors who were appointed between 1933 and 1945. Specific occurrences: 1: 27 Institute Directors were appointed between 1933 and 1945, 22 of whom were members of the NSDAP. 2: Five Jewish Institute Directors had to give up their posts in 1933 in Germany. Three more had to give up their posts in Austria and Czech Republic in 1938. 3: Ten Institute Directors were provisionally appointed in 1933. 4: 19 institute Directors were removed from office in 1945 and 1946. 5: Four Institute Directors who were appointed after 1933 were able to retain their posts after 1945. 6: 13 of the dismissed Institute Directors were allowed to take up office again after 1945. 7: Of the Institute Directors appointed between 1945 and 1963, four were Jewish or had Jewish ancestors. * This illustration does not show the normal Institute Directors change

**Fig. 5:**
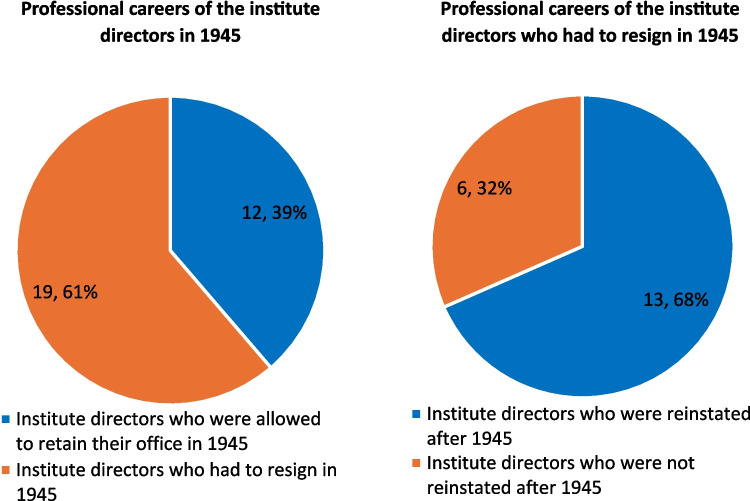
Review of the changes in institute management in the post war era–comparison of remaining and dismissed as well as reinstated and non-reinstated institute directors

There were a total of 137 changes of office in the period under review. Political and racial motives were identified for 28 changes of office. Most of them took place between 1933 and 1945/46, with a noticeable accumulation around the years 1933 and 1945 (Figs. 3 and 4). In the period from 1933 to 1945, there were 33 university pharmacological institutes: nine institute directors were in office without interruption over the entire period. In the remaining 18 university institutes, 27 institute directors were newly appointed during this period, 22 of them were members of the NSDAP (Fig. 4). In 1945 and 1946, 19 (61%) of the 31 institute directors were removed from office by the Allies for political motives (denazification). Of these, 13 (68%) were able to resume a position as institute director at another German-speaking pharmacological institute in the years after 1945 (Figs. 4 and 5). Between 1933 and 1938, a total of nine institute directors who were “non-Aryan” according to the ideology of National Socialism were expelled for racial motives. After 1945, there was only one Jewish institute director and three with Jewish ancestors in Germany until 1963 (Fig. 4).

**Fig. 6:**
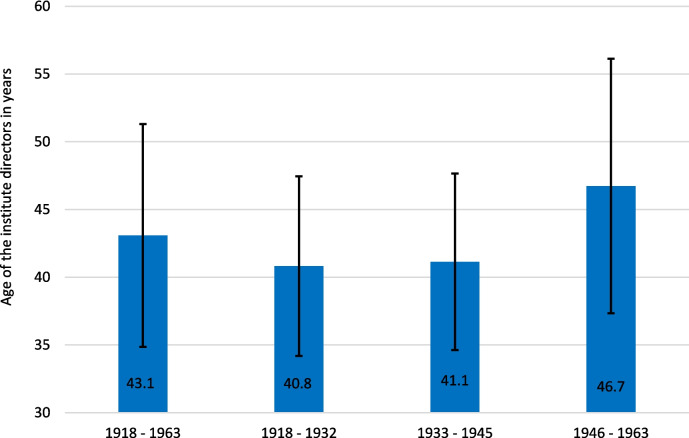
Average age of institute directors at the time of appointment for the entire observation period 1918–1963 and for individual time windows with standard deviation

Over the entire period under review, the institute directors were on average 43.1 years old when they took office. The youngest were 31 years old when they were appointed and the oldest was appointed head of institute at the age of 76. It is striking that the average age from 1946 to 1963, at 46.7 years, is significantly higher than in the previous periods (Fig. 6). This is caused by the above-mentioned re-appointment to another professorship.

During the interwar period, almost all institute directors were German, three were Austrians (Rudolf Gottlieb in Heidelberg, Julius Pohl in Breslau, Hermann Tappeiner in Munich), and one was Swiss (August Gürber in Marburg). This corresponded with a long tradition of close scientific exchange of German-speaking pharmacologists. Only one, Georg Joachimoglu, came from Greece, who was an extraordinary professor in Berlin. This picture remained nearly unchanged during the Nazi era. After 1945, Germans and Austrians separated again. Austrians, now again with their own citizenship, ran the Austrian university institutes (Hans Häusler in Graz, Adolf Jarisch in Innsbruck, and Heribert Konzett in Innsbruck). In Germany, only one Swiss ran a university institute after 1945 (Robert Domenjoz in Bonn).

Before 1933, 14% of the chair holders of German and Austrian pharmacology were Jewish—which was an astonishing growth for such a small population group (In 1933, around 0.77% of the German population was Jewish (Bundeszentrale für politische Bildung)). Before Weimar Republic, it was necessary being baptized and became catholic or protestant for a professorship. Only since 1919 did Jews have all civil rights and a full recognition as citizens took place. This included the possibility to become a professor at a university. This changed completely after 1933. There were no Jewish institute directors during this period anymore. As mentioned above, in the period 1945–1963 only four of the university pharmacological institutes considered were headed by an institute director of Jewish faith or decent.

### NSDAP memberships and memberships in other Nazi organizations

**Fig. 7:**
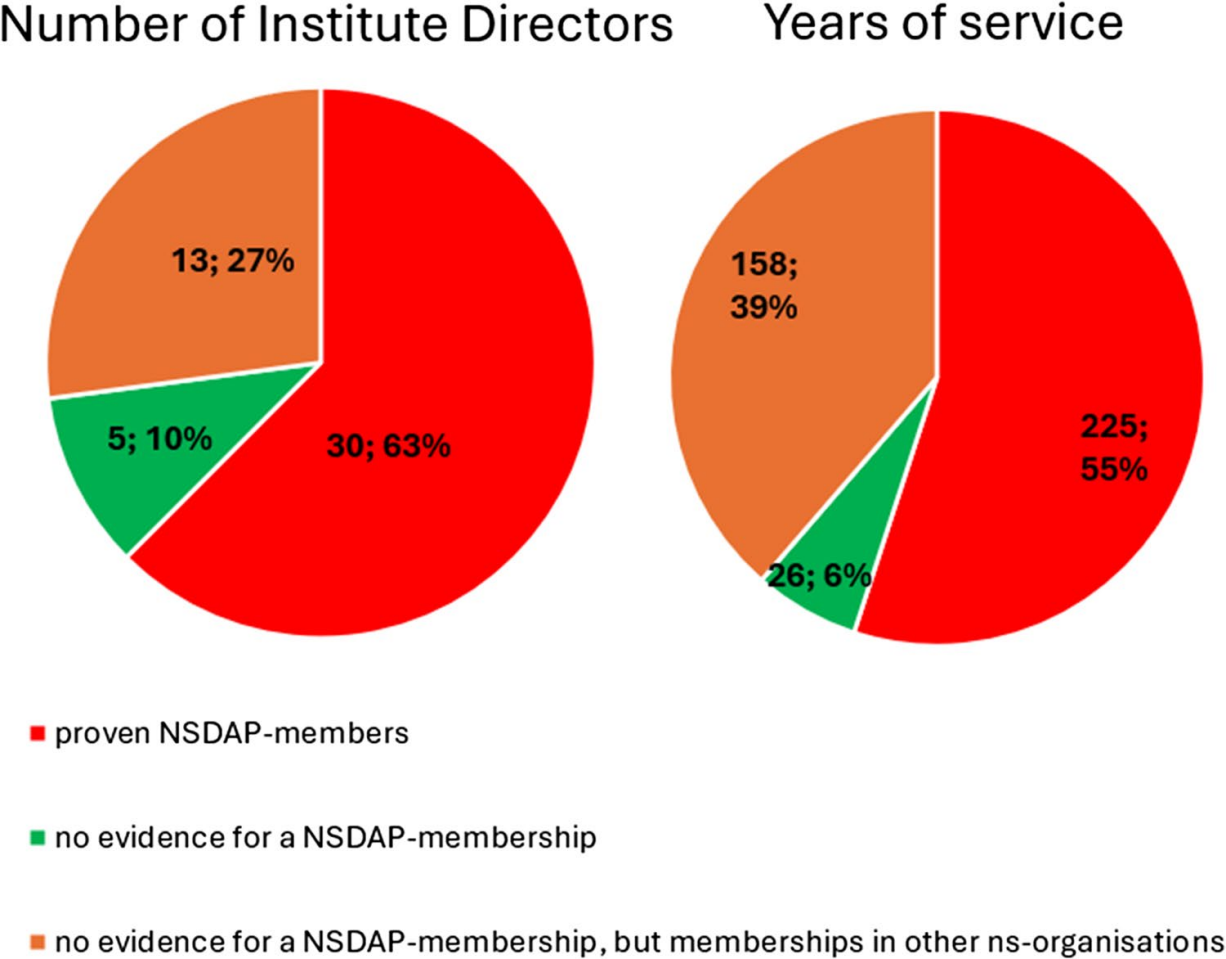
Assessment of NSDAP memberships of institute directors in the period of National Socialism. By number of persons (left) and by years of service of the institute directors (right)

Of the 48 institute directors working under National Socialism, 30 (63%) were provable members of the NSDAP. No evidence for a NSDAP membership could be found for five pharmacologists (10%). For the remaining 13 institute directors (27%), there is no evidence for a NSDAP membership, but they were members of other National Socialist organizations (Fig. 7).

**Fig. 8:**
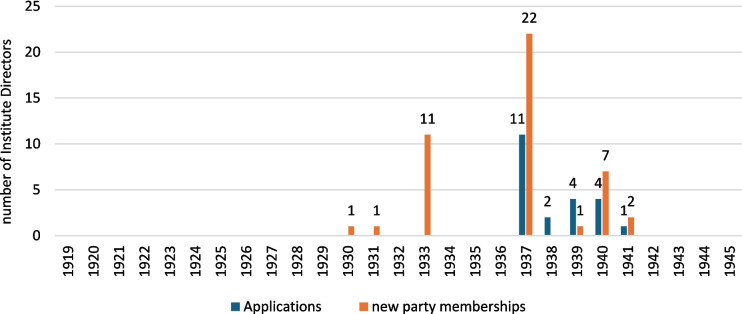
Overview of applications for membership and membership of the DAP/NSDAP from institute directors in the period 1919–1945

Of the persons with confirmed NSDAP membership, two joined the NSDAP before 1933, eleven others joined in 1933, and the remaining persons joined between 1937 and 1941 (Fig. 8).

**Fig. 9:**
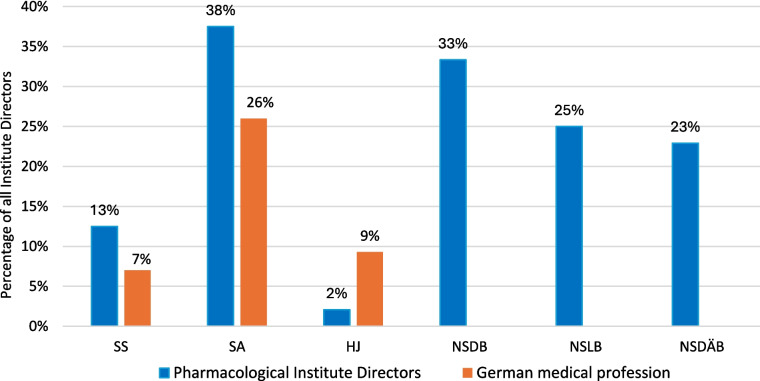
Proportion of memberships of institute directors in National Socialist organizations (not NSDAP) and comparison with proportions of memberships of German doctors according to Kater (2000)

**Fig. 10:**
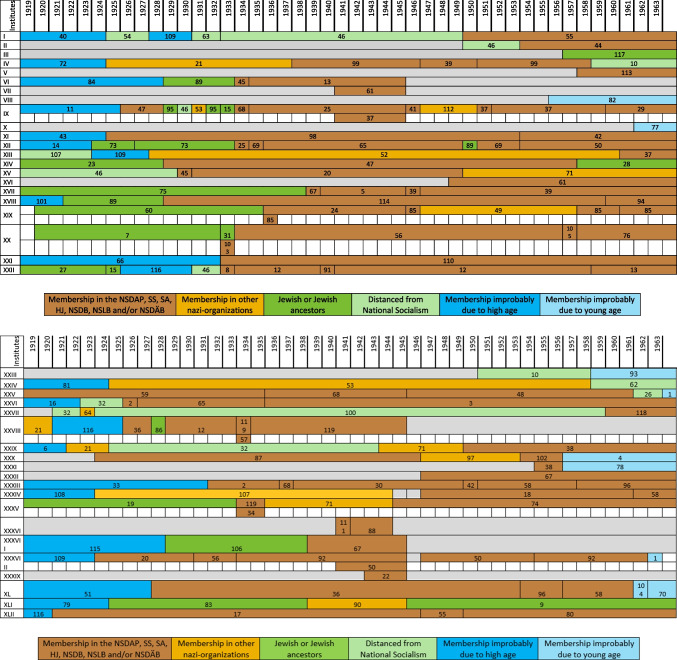
Timeline for the directors of the individual university pharmacological institutes. The institutes for the numbers (I to XLII) are listed in Table 2. The pharmacologists for the numbers (1 to 119) are listed in Table 3. If more than one pharmacologist was head of an institute in the same year, they are listed one below the other

The major part of the institute directors who worked under National Socialism were also active or supporting members of other National Socialist associations. The most common associations were the *Sturmabteilung* (SA) (38%), the NS-Lecturers’ association (*NS-Dozentenbund*) (33%), the NS-Teachers’ Association (*NS-Lehrerbund*) (23%), the NS-Medical Association (*NS-Ärztebund*) (23%), the *Schutzstaffel* (SS) (13%), and the Hitler Youth (*Hitlerjugend*) (HJ) (2%) (Fig. 9). A list of all institute directors by name who were members of the NSDAP, SS, and/or SA is shown in Table 3 and Fig. 10.

**Table 3:**
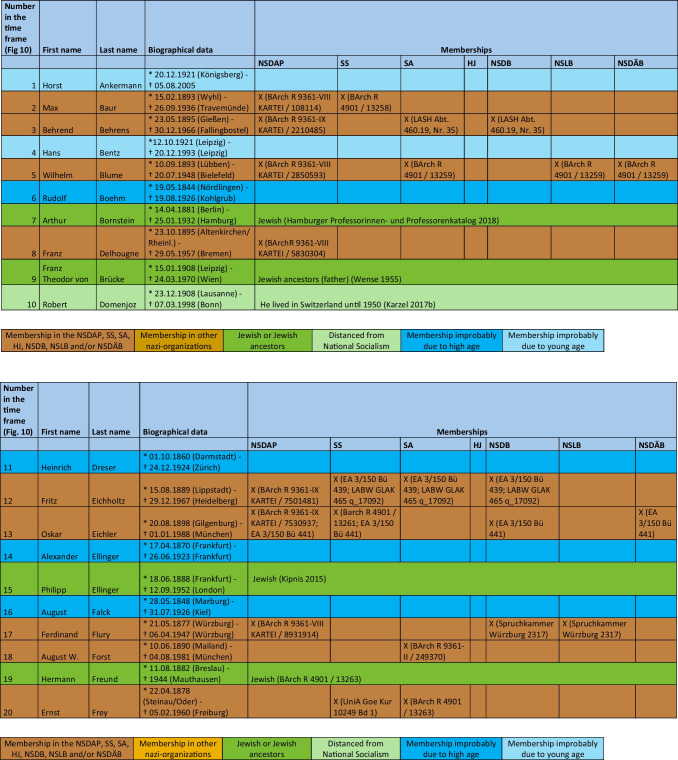

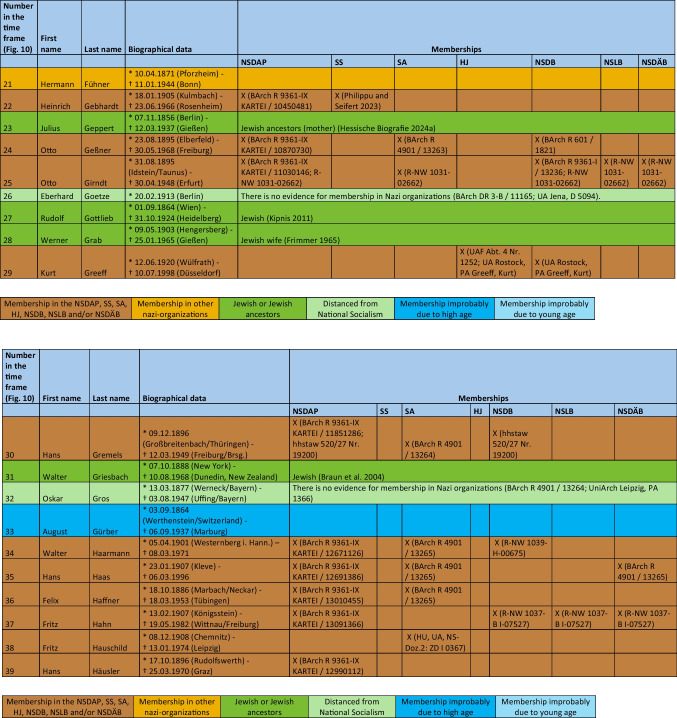

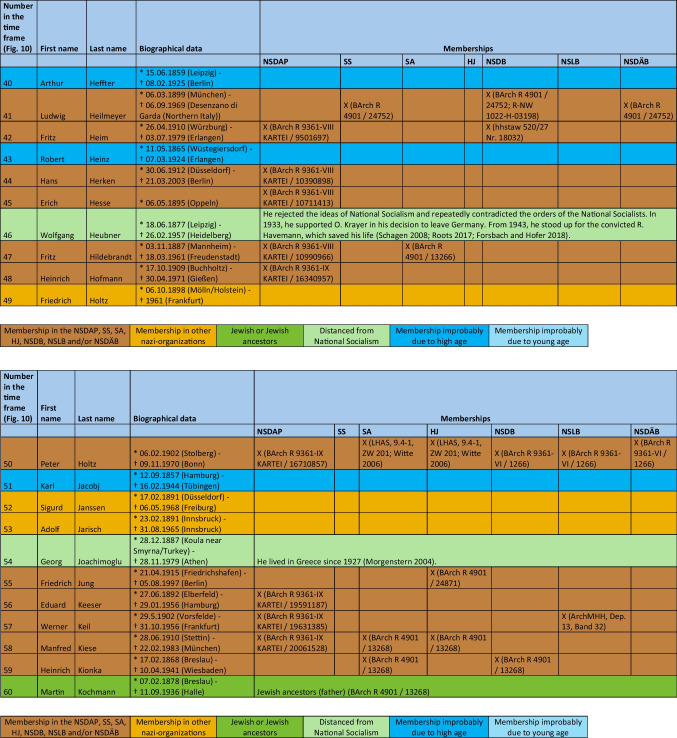

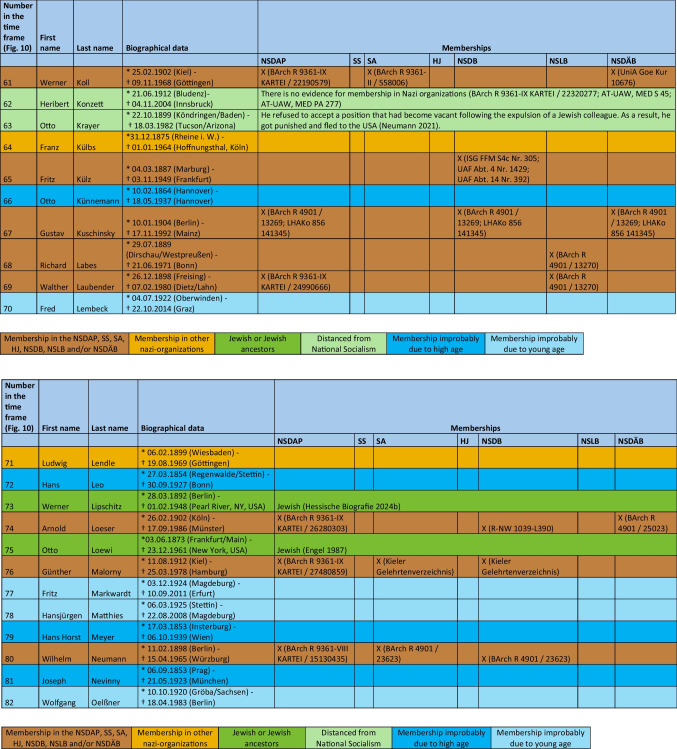

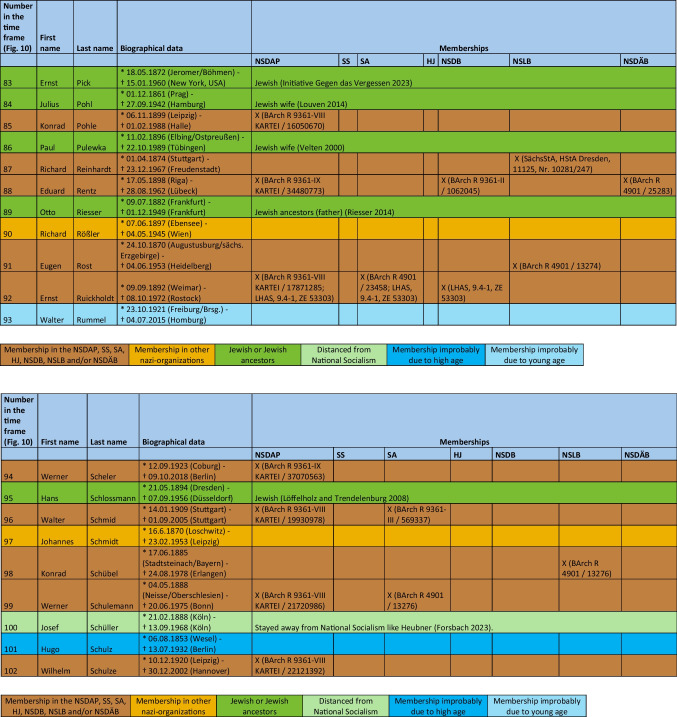

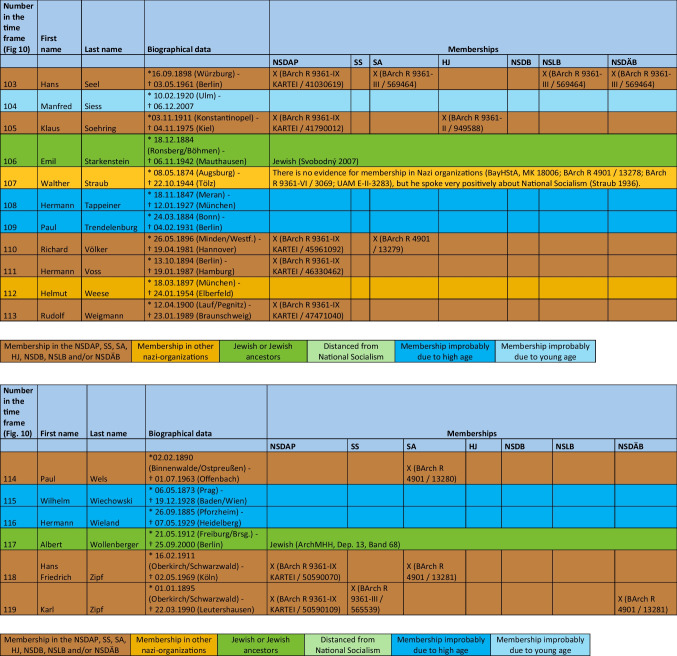
List of all institute directors and their memberships in the NSDAP, SS, SA, HJ, NSDB, NSLB and NSDÄB

### Military service and research in the second world war

**Fig. 11:**
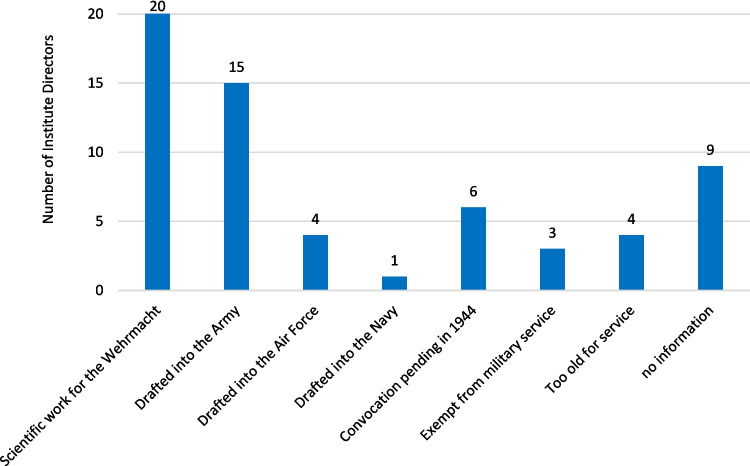
Military activities of the Institute Directors in World War II

Twenty of the institute directors who worked under National Socialism conducted scientific research for the German military. Nineteen pharmacologists were deployed in the war. Of these, 15 were in the Army (*Wehrmacht*), four in the Airforce (*Luftwaffe*), and one in the Navy (*Marine*). One institute director worked for both the Army (*Wehrmacht*) and the Airforce (*Luftwaffe*). Six others had not yet been called up in 1944. Three were exempt from military service and four were already too old. No information could be found on nine institute directors (Fig. 11).

## Discussion

### Number of institute directors and reasons for change of office

With two exceptions, all politically motivated changes of office of individual institute directors took place between 1933 and 1946. One exception occurred in 1921 with the introduction of a new law which, among other issues, stipulated the maximum age of university lecturers (Siegmund et al. 2004). The other exception (in 1957) was Friedrich Holtz, the then director of the institute in Halle, who was threatened with disciplinary proceedings because of political statements, which is why he fled the German Democratic Republic (Deutsche Demokratische Republik, DDR) (Giessler and Giessler 2004). The many politically motivated changes from 1933 onwards can be explained by the “Act on the Restoration of the Professional Civil Service” (“Gesetz zur Wiederherstellung des Berufsbeamtentums”) introduced by the National Socialists on April 7, 1933. With the help of this law, persons considered “non-Aryan” and political opponents were gradually removed from the civil service. In particular, these were the heads of the university pharmacological institutes in Breslau (Otto Riesser) Düsseldorf (Philipp Ellinger), Frankfurt (Werner Lipschitz), Hamburg (Walter Griesbach), and Münster (Hermann Freund) (Lindner and Lüllmann 1996). There were exceptions until 1935, for example, for persons who fought in the First World War (“Frontkämpferprivileg”) (Beddies et al. 2014). For this reason, Martin Kochmann, head of the Institute of Pharmacology in Halle, who was considered a “non-Aryan,” was able to retain his post until 1935. He resigned due to the convention of speech “circumstances of the time.” In 1936, he was accused by the Gestapo of promoting anti-state and highly treasonous endeavors and was therefore imprisoned. He is said to have committed suicide in prison in the same year (Giessler and Giessler 2004). From 1938 onwards, there were further expulsions in the territories newly annexed to National Socialism, such as Austria and the Czech Republic. In particular, the heads of the university pharmacological institutes in Graz (Otto Loewi), Prag (Emil Starkenstein), and Wien (Ernst Peter Pick) were expelled (Lindner and Lüllmann 1996). Loewi was imprisoned in Graz in 1938 and had to buy his freedom with the prize money from his Nobel Prize. He first emigrated to England and then took up a position in New York, where he remained until his death (Lindner and Lüllmann 1996; Lembeck et al. 2004). His expulsion shows how consistently and irrespective of previous scientific successes and ranks, this law was applied (Engel 1987).

Mention should also be made here of the pharmacologists who were not expelled from their posts after 1933 because they had none, but who nevertheless actively refused to play an active role in National Socialism. The best-known example of this is Otto Krayer, who refused the appointment to the position in Düsseldorf that had become vacant following the expulsion of Philipp Ellinger and emigrated first to England and then to the USA (Neumann 2021). Another example was Paul Pulewka, a pharmacologist in Tübingen, whose wife was Jewish. Therefore, he and his family fled to Ankara in 1935 (Velten 2000). A full list of all institute directors who were victims of the National Socialism shows Table 4. In their book, Konrad Löffelholz and Ullrich Trendelenburg (2008) reappraised the fates of persecuted German pharmacologists. On this basis, Mispagel and Seifert (2024) examined the consequences of the expulsions in their scientific work.

**Table 4:**
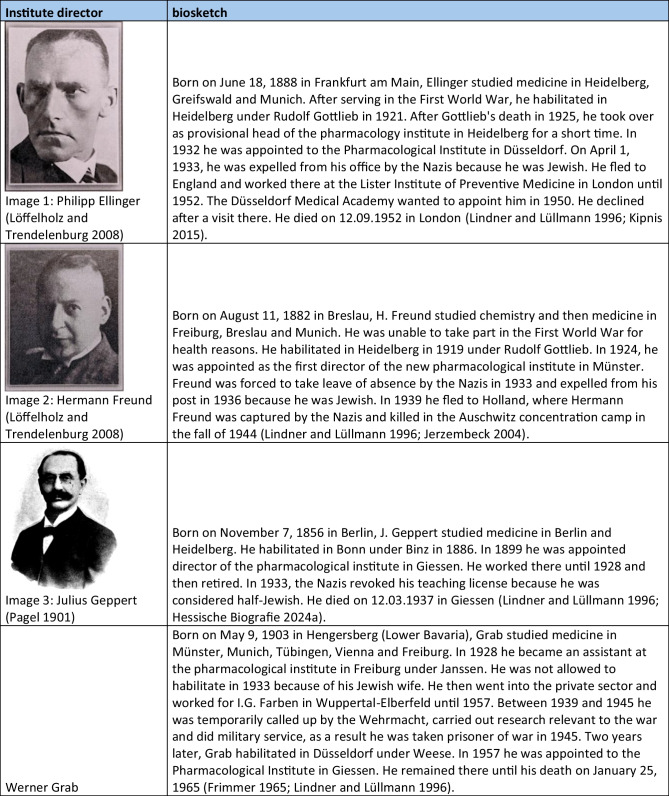

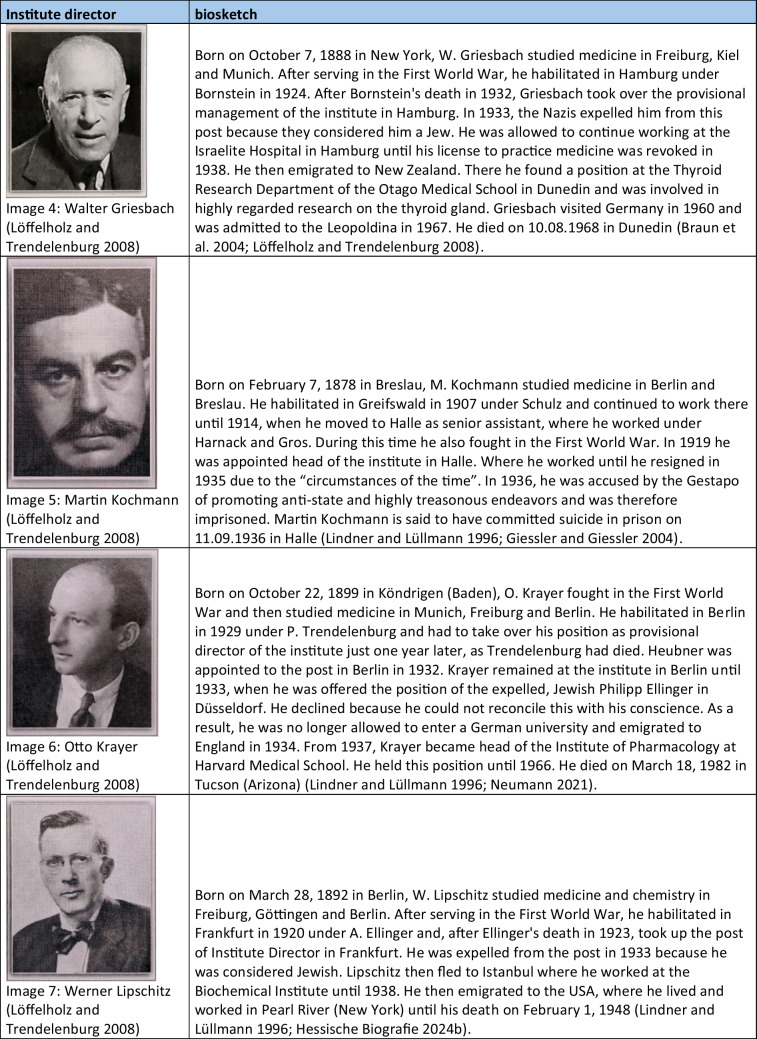

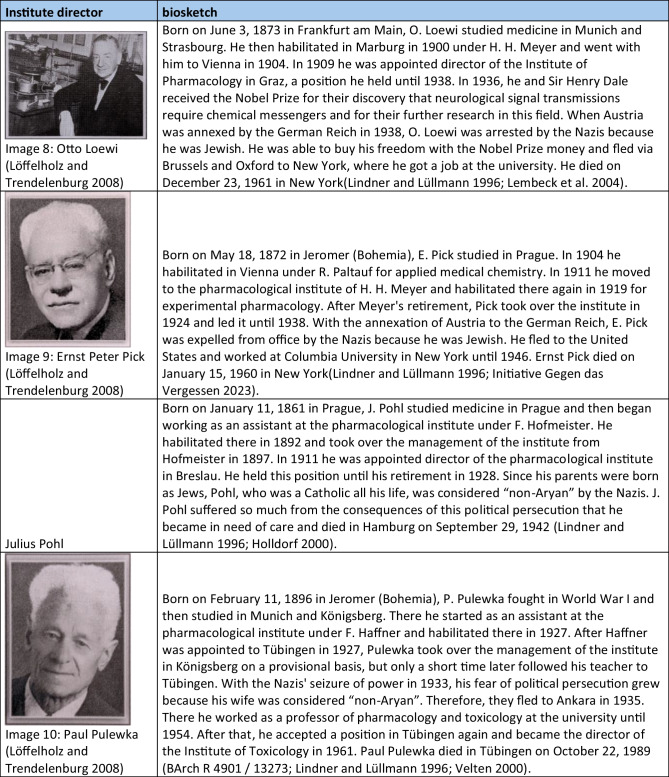

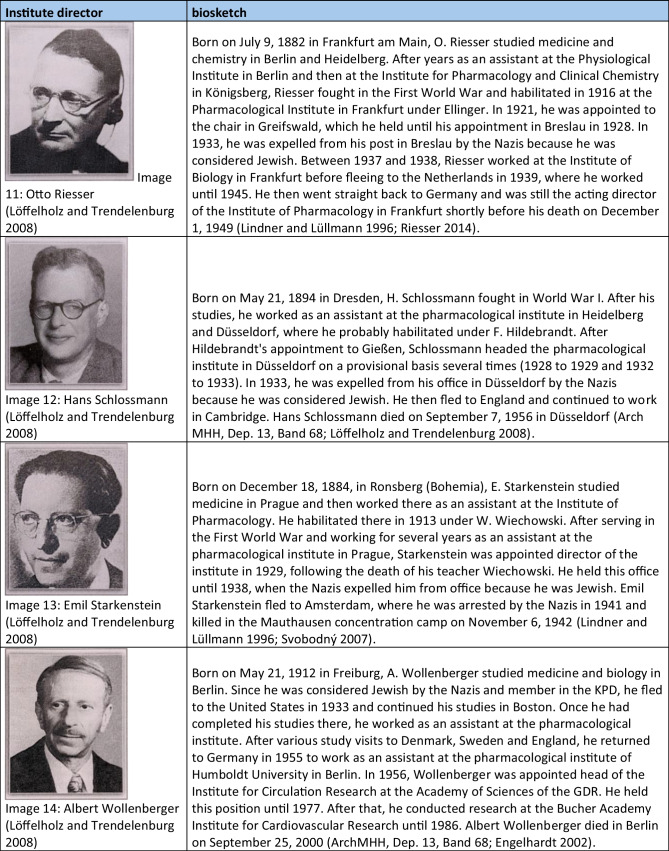
Short portfolios of institute directors who were victims of national socialism. The images were gratefully provided by Dr. Schrör Verlag (except for picture 3)

No politically motivated changes could be found between 1940 and 1943. This can be explained by the fact that all the institute directors who fell under the racist law had already been expelled and their positions filled with Aryan scientists. This could also indicate that these newly appointed institute directors were loyal or conformist to National Socialism. Examples of this include the Würzburg institute`s director Ferdinand Flury, who was already conducting research into warfare agents before 1933 had been a member of various National Socialist associations since 1934 (Spruchkammer Würzburg 2317), joined the NSDAP in 1937 (BArch R 9361-VIII KARTEI/8,931,914), and continued with poison gas research for the German military in Würzburg (Schmaltz 2017). Similarly, the pharmacologist Heinrich Gebhardt, was specially appointed from Munich to the newly founded Reich University of Strasbourg and was a member of the NSDAP and the SS, among others (Möhler 2020).

Gustav Kuschinsky was appointed as a “Reichsprofessor” to the position in Graz that had become vacant following O. Loewi’s imprisonment due to his many years of work abroad in Shanghai (Scholz 2017). He was also a member of the NSDAP, NSDB, and NSDÄB (BArch R 4901/13,269). A year later, in 1939, he was appointed to the post in Prague that had become vacant following the expulsion of the Jewish director of the institute, Emil Starkenstein. He remained there until 1945; from 1946, he became director of the institute in Mainz until 1972. For German Pharmacology and Toxicology scientists and many physicians still alive, the name Gustav Kuschinsky is associated with the textbook,"Kuschinsky-Lüllmann.” He used the Mainzer Frühjahrstagung der DGPT, the largest meeting of pharmacologists, toxicologists and clinical pharmacologists in Europe, to ascertain his political influence in pharmacology and toxicology in German-speaking countries until in the early 1990s (Scholz 2017). A full list of pharmacologists who benefited from and supported the racially motivated expulsions from 1933 onwards is shown in Table 5.

**Table 5:** Short portfolios of institute directors who benefited from national socialism

Wilhelm Blume (1893–1948)
Born on September 10, 1893 in Lübben (Spreewald), W. Blume studied medicine in Berlin and Würzburg. After his studies and military service in World War I, he began working as an assistant at the 1 st Medical Clinic in Berlin in 1922. In 1923, he took a job as an assistant at the Institute for Physical Chemistry in Berlin and moved to Freiburg in 1924. In 1925, he became an assistant at the Institute of Pharmacology in Bonn under H. Fühner, with whom he habilitated in 1929. In 1933, he joined the SA. He was also a member of the NSLB, NSDÄB, RLB and NSV (BArch R 4901/13,259). Blume worked in Bonn until 1936, when he left for Canton, China, for three years. For his work in China, he was appointed “Reichsprofessor,” which entitled him to the next available professorship in pharmacology (Lembeck et al. 2004). In 1937, he joined the NSDAP (BArch R 9361-VIII KARTEI/2,850,593). When he returned from China in 1939, he accepted the position as head of the pharmacological institute in Graz. This position had become vacant because the Jewish O. Loewi was forced out of office by the Nazis in 1938 and G. Kuschinsky took over the position provisionally. Wilhelm Blume had to give up the position in Graz in 1945 and died a little later on July 20, 1948 in Bielefeld (Lindner and Lüllmann 1996)
**Oskar Eichler (1898–1988)**
Born on 20 August 1898 in Gilgenburg (East Prussia), O. Eichler fought in the First World War and then volunteered for the East Border Police. During this time, he was a member of the Pan-German League and the German-Völkisch Freedom Party (Barch R 4901/13,261). After studying medicine in Königsberg and Munich, he first became an assistant at the pharmacological institute in Königsberg under H. Wieland. Shortly after that, he transferred to the Institute for Internal Medicine in Heidelberg, only to go to the pharmacological institute in Düsseldorf under F. Hildebrandt in 1926. When Hildebrandt was appointed to the pharmacological chair in Gießen in 1928, Eichler followed him and habilitated there in 1930. In Gießen, he became a sponsoring member of the SS and the Dozentenführer (Barch R 4901/13,261). In 1933, he joined the NSDAP (BArch R 9361-IX KARTEI/7,530,937). In 1934, he was appointed to the chair of pharmacology in Breslau. This position had become vacant because its Jewish incumbent, O. Riesser, had been expelled by the Nazis in 1933. E. Hesse held the position provisionally until Eichler accepted the offer. Eichler, who had become an active member of the SS, NSDB, NSDÄB and NSV (EA 3/150 Bü 441), worked for the Wehrmacht as a consultant pharmacologist in the invasion of Poland from 1939 and then for the medical inspectorate, as well as a consultant pharmacologist in the reserve army in military district VIII (ArchMHH, Dep. 13, Band 124; Eichler 2011). When the University of Breslau was closed by the Allies in 1945, Eichler lost his position and was interned until 1947 (Grüttner 2004). After his release, he was allowed to continue working in rooms of the surgical clinic in Heidelberg until he was appointed to the chair of clinical pharmacology in Heidelberg in 1955. In 1950, he became an Editor of “Handbooks of Experimental Pharmacology” (Eichler 2014) In 1958, he was appointed to the Chair of Pharmacology in Heidelberg, which he held until his retirement in 1968. Oskar Eichler died in Munich on January 1, 1988 (Lindner and Lüllmann 1996)
**Heinrich Gebhardt (1905–1966)**
Born on January 18, 1905 in Kulmbach, H. Gebhardt first studied chemistry and then medicine in Munich. In 1933, he joined the SS. One year later, he became an assistant at the pharmacological institute in Munich under Straub. In 1937, he joined the NSDAP. Gebhardt habilitated under Straub in 1939. In the meantime, he had risen to the rank of SS-Untersturmführer in the SS, and he the head of the lecturers at the University of Munich, and the leader of the NSD-Gaudozentenbund for Munich and Upper Bavaria. He was also a member of the NSDÄB and NSV (Möhler 2020). From 1939 he taught at the gas protection school in Celle. In 1942 the Nazis appointed him head of the pharmacological institute of the Reich University in Strasbourg. From 1940 he published the textbook “Grundriß der Pharmakologie, Toxikologie (Wehr-Toxikologie) und Arznei-Verordnungslehre”. After the destruction of the institute in Strasbourg in 1944, Gebhardt fled back to Germany, where he probably settled as a general practitioner in Rosenheim from 1949. Heinrich Gebhardt died there on June 23, 1966 (Möhler 2020)
**Otto Geßner (1895–1968)**
Born on August 23, 1895 in Elberfeld, O. Gessner first studied mathematics and natural sciences in Münster and later medicine in Marburg and Strasbourg. After his studies and military service in World War I, he worked as a resident physician in Lippischen until 1923. In 1923, he began working as an assistant at the Institute of Pharmacology in Marburg under A. Gürber and from 1931 under M. Baur. There he was habilitated in 1927. Geßner joined the SA in Marburg (BArch R 4901/13,263). In 1935, the Jewish M. Kochmann was expelled by the Nazis from his post as head of the pharmacological institute in Halle. Gessner accepted the vacant position and joined the NSDAP in the same year (BArch R 9361-IX KARTEI/10,870,730). He was also a member of the NSDB (BArch R 601/1821). He was not called up for military service because he was entrusted with defense-related scientific tasks (ArchMHH, Dep. 13, Band 124). In 1945, he was removed from office by the Allies and then settled in Bielefeld as a practicing physician. Otto Gessner died on May 30, 1968 in Freiburg (Giessler and Giessler 2004; Catalogus Professorum Halensis 2024b)
**Otto Girndt (1895–1948)**
Born on August 31, 1895 in Idstein (Taunus), O. Girndt studied medicine in Tübingen and fought in World War I. What he did in the following years is unknown. In 1926 he began as an assistant at the pharmacological institute in Frankfurt under W. Lipschitz. He habilitated there in 1933. In the same year, he joined the NSDAP (BArch R 9361-IX KARTEI/11,030,146) and the SA and provisionally took over the management of the institute from Lipschitz, who, as a Jew, was expelled from his position. In 1934, 1 year later, Girndt was appointed to the professorship in Düsseldorf against the will of the local faculty (BArch R 9361-II/295,622). This professorship had also become vacant due to the expulsion of the Jewish institute Director P. Ellinger. In Düsseldorf, he became “Dozentenschaftsleiter” in 1934 and “Gaudozentenbundführer” in 1936 (BArch R 9361-I/13,236). He was also a member of the NSDÄB, NSLB, NSDB, NSV and RLB (R-NW 1031–02662). As a consultant pharmacologist for the armed forces, he was involved in the France and Russia campaigns (BArch R 9361-VI/847). From 1939, his institute in Düsseldorf became a branch of the Military Medical Academy and the Army Ordnance Office, for which he conducted pharmacological nerve gas research on Tabun (Schmaltz 2006). Due to his many obligations, he was hardly ever in Düsseldorf from 1940, which is why Fritz Hahn took over as acting director. Otto Girndt was removed from office by the Allies in 1945. He contracted tuberculosis and died of it on April 30, 1948 in Erfurt (Lindner and Lüllmann 1996)
**Wolfgang Heubner (1877–1949)**
Born on June 18, 1877 in Leipzig, W. Heubner studied medicine in Göttingen, Marburg and Strasbourg. After that, he became an assistant at the pharmacological institute in Strasbourg under O. Schmiedeberg in 1903. In 1905 and 1906 he paused his work in Strasbourg to study chemistry in Munich und Zürich. One year later, he habilitated in Strasbourg under O. Schmiedeberg. In 1907, Heubner began working as an assistant at the Institute of Pharmacology in Berlin under A. Heffter. One year later, he became a call to the chair in Göttingen, which he holds until 1929 (BArch R 4901/13,266; BArch R 9361-IX KARTEI/7,530,937). In 1911, he was involved in the founding of the first pharmaceutical commission in Germany (Roots 2017). Heubner had been working for the military from time to time since 1896, including throughout the First World War. In 1929, he takes a call to Düsseldorf, only to go to Heidelberg one year later. Heubner holds the chair in Heidelberg for two years until he became a call to Berlin in 1932 (BArch R 4901/13,266). In the same year he became a member of the DPhG board and a year later its acting director. In 1936, he became a member of the Leopoldina (Roots 2017). The only known membership in a Nazi organization was in the Reichsluftschutzbund (Kessel 2008). He repeatedly criticized National Socialism and tried to protect his staff at the institute from it (Schagen 2008). Heubner also conducted research for the Wehrmacht, including on combat gases (BArch R 26-III/80,769). He was accused of at least knowing about human experiments in concentration camps. Heubner denied this (Herken 1999). In 1950, he took over the Institute of Pharmacology at the newly founded Free University of Berlin and became Dean of the Faculty of Medicine. He remained there until his retirement in 1953. In the following years, Heubner received many awards. These included the Grand Cross of the Order of Merit of the Federal Republic of Germany in 1952, the Paracelsus Medal of the German medical profession in 1954 and the Schmiedeberg Plaque of the DPhG in 1956. Wolfgang Heubner died on February 26, 1957 in Heidelberg (Roots 2017)
**Eduard Keeser (1892–1956)**
Born on June 27, 1892 in Elberfeld, E. Keeser studied medicine in Tübingen, Bonn and Berlin. After serving in World War I and briefly working as an assistant at the University of Berlin’s Medical Clinic I, he began working as an assistant at the Institute of Pharmacology in Berlin under A. Heffter in 1923. He was habilitated there in 1927. One year later, he became a senior government councilor in the Department of Pharmacology at the Reich Health Office in Berlin (Hennighausen and Paegelow 2004). In 1930, he accepted the professorship in Rostock. When the Jewish director of the Institute of Pharmacology in Hamburg, W. Griesbach, was dismissed from his post by the Nazis in 1933, Keeser took over the chair. He became Dean of the Medical Faculty from 1934 until 1938 and, in parallel, Medical Director of the UKE until 1936 (Braun et al. 2004, ). He joined the NSDAP in 1937 (BArch R 9361-IX KARTEI/19,591,187). During the 1939 invasion of Poland, he served as an expert in chemical weapons (BArch R 4901/23,085). After that, he served the Wehrmacht as a consulting pharmacologist in the reserve army in military district 10 (ArchMHH, Dep. 13, Band 124). From 1941 to 1945, Keeser was rector of the University of Hamburg. After Keeser underwent denazification proceedings by the Allies in 1945, he was allowed to continue to head the Institute of Pharmacology in Hamburg until his death on January 29, 1956 (Braun et al. 2004; Catalogus Professorum Rostochiensium 2022)
**Werner Koll (1902–1968)**
Born on February 25, 1902 in Kiel, W. Koll studied chemistry and medicine in Kiel and Munich. After that, he became an assistant at the pharmacological institute in Berlin under P. Trendelenburg in 1929. In 1932, he transferred to the pharmacological institute in Kiel under F. Külz. In 1933, he joined the SA (BArch R 9361-II/558,006) and in the following years the NSDÄB, NSV and RLB (UniA Goe Kur 10,676). When Külz was appointed to the pharmacological chair in Frankfurt in 1935, Koll followed him and habilitated there in the same year. The chair in Frankfurt had become vacant because its Jewish incumbent W. Lipschitz was expelled by the Nazis in 1933. O. Girndt and W. Laubender held the position provisionally until Külz accepted the call. W. Koll officially joined the NSDAP in 1937 (BArch R 9361-IX KARTEI/22,190,579). In 1939, he was officially called up to work at the Military Medical Academy in Berlin. When the Nazis wanted to expand the Medical Academy in the conquered Danzig in 1940, they appointed Koll as head of the new pharmacological institute in Danzig. Under Koll, the institute in Danzig was used as a branch of the Military Medical Academy and the Army Ordnance Office for pharmacological nerve gas research (Schmaltz 2006). In addition, from 1940, Koll was a consultant pharmacologist in the reserve army in military district 20 (ArchMHH, Dep. 13, Band 124). When Danzig was conquered by the Allies in 1945, he fled to Kiel and later to Kappeln. As a member of the founding board of trustees, he was appointed director of the pharmacological department of the medical research institute of the Max Planck Society in Göttingen in 1947. In the following years, Koll was, among other things, chairman of the Drug Commission of the German Medical Association, a member of the Military Medical Advisory Board of the Federal Ministry of Defense and an advisor to the WHO. He was awarded the Paracelsus Medal of the German Medical Association in 1965 (Wellhöner 2017). He held his position with the Max Planck Society until his death in Göttingen on November 9, 1968 (Lindner and Lüllmann 1996)
**Fritz Külz (1887–1949)**
Born on March 4, 1887 in Marburg, F. Külz studied medicine in Freiburg, Munich, Marburg, and Berlin. After his studies and military service in World War I, he began working as an assistant at the Institute of Pharmacology in Leipzig under H. Fühner in 1919. There he habilitated in 1922. From 1926, he took over the pharmacological chair in Kiel. When the Jewish W. Lipschitz was expelled from his position as head of the pharmacological institute in Frankfurt by the Nazis in 1933, O. Girndt and W. Laubender represented him on a provisional basis until Külz accepted the call to the vacant position in 1935. There is no information that Fritz Külz was a member of the Nazi Party. He was a member of the NSDB in Frankfurt (ISG FFM S4c Nr. 305; UAF Abt. 4 Nr. 1429; UAF Abt. 14 Nr. 392). There are also no sources about his activities for the armed forces. Külz headed the institute in Frankfurt until his death on November 3, 1949 in Frankfurt. The Fritz Külz Prize, the DGPT junior scientist award, is still financed with the financial proceeds from his research (Kieler Gelehrtenverzeichnis; Lindner and Lüllmann 1996)
**Gustav Kuschinsky (1904–1992)**
Born on January 10, 1904 in Berlin, G. Kuschinsky studied medicine in Berlin, Tübingen, Marburg and Innsbruck. After his studies, he began working as an assistant at the Institute of Pharmacology in Berlin under P. Trendelenburg, O. Krayer and finally W. Heubner, under whom he habilitated in 1933. After that, he went to Shanghai until 1936 to work at the state-run Tongji University, where he co-founded the China chapter of the German Medical Association. For his work in China, he was appointed “Reichsprofessor” (Reich Professor), which entitled him to the next vacant professorship in pharmacology (Lembeck et al. 2004). When he returned to the institute in Berlin in 1937, he joined the NSDAP (BArch R 4901/13,269), as well as the NSDÄB, NSDB and NSV (LHAKo 856 141,345). When the Jewish O. Loewi was expelled by the Nazis from his position as head of the pharmacological institute in Graz in 1938, Kuschinsky took over the position provisionally. A short time later, the Jewish director of the institute, E. Starkenstein, was also expelled in Prague. Kuschinsky accepted the appointment to the vacant position and headed the institute in Prague until he fled from the Red Army to Erlangen and later to Wiesbaden in 1945 (Scholz 2017). He was not drafted during this time because he was entrusted with defense science tasks (ArchMHH, Dep. 13, Band 124). In 1946, Kuschinsky was appointed to the newly founded Institute for Pharmacology in Mainz. He received several awards in the following years, including the Schmiedeberg Medal from the DGPT in 1982, of which he also became an honorary member in 1970. The textbook “Kurzes Lehrbuch der Pharmakologie” by Kuschinsky and Lüllmann is still well-known among German physicians today (Scholz 2017). Gustaf Kuschinsky retired in Mainz in 1972 and died in Mainz on November 17, 1992(Lindner and Lüllmann 1996)
**Ludwig Lendle (1899–1969)**
Born on February 6, 1899 in Wiesbaden, L. Lendle fought in World War I. After that, he studied medicine in Frankfurt, Gießen and Freiburg. In 1923, he began working as an assistant at the Institute of Pharmacology in Kiel and a year later moved to Leipzig to work at the institute under O. Gros. There he habilitated in 1928. In 1935, he went to work for Heubner in Berlin, only to accept an appointment to the chair in Münster a year later (Schmidt and Lendle 1985). This chair had become vacant because its Jewish incumbent, H. Freund, had been expelled. Lendle was a member of the NSV (UniArch Leipzig, PA 189). There is no data on his membership of the NSDAP or other National Socialist organizations. In 1939, he was drafted to work in the pharmacological-toxicological department of the Military Medical Academy in Berlin. In this context, his institute in Münster became a branch of the Military Medical Academy and the Army Ordnance Office for pharmacological nerve gas research. Among other things, he conducted research with the nerve gases Soman, Serin, and Tabun (Schmaltz 2006). He worked for the Wehrmacht as a consulting pharmacologist (rank: Oberfeldarzt der Reserve) in the Ersatzheer (substitute army) in military district 4 (BArch R 9361-II/630,397). In 1943, Lendle was appointed to the chair in Leipzig. There he continued his research and rebuilt the institute after its destruction in 1945. In 1948, he accepted the offer of the professorship in Göttingen, which he held until his retirement in 1969. Ludwig Lendle died a little later on August 19, 1969 in Göttingen (Lindner and Lüllmann 1996)
**Eduard Rentz (1898–1962)**
Born on May 17, 1898 in Riga (Latvia), E. Rentz fought in World War I. After that, he first studied at the German University in Dorpat (Estonia) and later medicine in Riga. As early as 1926, he spent a semester in Munich. In 1932, he habilitated at the Institute of Pharmacology in Riga. In 1940, Rentz was invited by the head of the Institute of Pharmacology in Munich, Straub. He accepted the offer and emigrated to Germany. There he immediately joined the NSDAP, as well as the NSDÄB, NSDB, and NSV (BArch R 9361-II/1,062,045; BArch R 4901/25,283). At that time, it was already planned that he would take over the position in Posen. In 1942, the Nazis appointed him head of the pharmacological institute of the Reich University in Posen (BArch R 4901/25,283). This position had previously been held by the anatomist H. Voss (BArch R 4901/13,279). Rentz was called up for military service in 1945, but quickly managed to be taken prisoner. After the war, he worked temporarily as a translator in Greifswald and as a pharmacologist for the company Schwabe in Leipzig. In 1948, he was appointed as a professor in Kiel, where he worked under Behrens, until his retirement in 1960. Eduard Rentz died on 28.08.1962 in Lübeck (Kieler Gelehrtenverzeichnis; Lindner and Lüllmann 1996)
**Richard Rössler (1897–1945)**
Born in June 7, 1897 in Ebensee (Upper Austria), R. Rössler fought in World War I and then studied medicine in Innsbruck. In 1923, he began working there as an assistant at the Institute for General and Experimental Pathology. Just one year later, he went to Loewi’s pharmacological institute in Graz, where he probably met E. Pick. Shortly afterwards, Pick was offered the chair of pharmacology in Vienna, and Rössler followed him there in 1924. He habilitated there in 1931 and became the first assistant at the institute. When Pick was forced out of office by the Nazis in 1938 because he was Jewish, Rössler took over the position. In the same year, he became a member of the NSDAP and the RLB (BArch R 9361-II/845,018). He was never drafted into military service because he was entrusted with defense-related scientific tasks (ArchMHH, Dep. 13, Band 124). Richard Rößler held the office in Vienna until he died on May 4, 1945 in Vienna from injuries of unknown origin (Hubenstorf 1898)
**Konrad Schübel (1885–1978)**
Born on June 27, 1885 in Stadtsteinach (Bayern), K. Schübel studied chemistry in Würzburg and medicine in Würzburg and München. In 1907, he began as an assistant at the Institute for chemistry in Würzburg, where he doctorated in 1909. One year later, Schübel switched to the pharmacology Institute in Würzburg. In 1913, he switched again, now to the Institute for Animal physiology in Munich. After 4 years fighting in World War I, he doctorated again in Munich 1920. Schübel then returned to the pharmacological institute in Würzburg, where he was habilitated under F. Flury in 1921. Just 3 years later, in 1924, he was appointed to the chair in Erlangen, after the death of the previous chairholder R. Heinz. In 1934 Schübel joined the NSLB (BArch R 4901/13,276). Memberships in other NS organizations or the NSDAP are unknown. In Erlangen, he worked on combat gases (Brune et al. 2004) and conducted research for the Wehrmacht (ArchMHH, Dep. 13, Band 124). Schübel stayed loyal to his position in Erlangen until his retirement in 1953, despite numerous appointments to other positions. Konrad Schübel died in Erlangen on August 8, 1978 (ArchMHH, Dep. 13, Band 31)
**Hans Seel (1898–1961)**
Born on September 16, 1898 in Würzburg, H. Seel fought in World War I and remained in the Freikorps Epp in Munich until 1920. In 1921, he joined the German Reichs-Volksschutz and Trutzbund and in 1923 transferred to the SA (BArch R 9361-III/569,464). Meanwhile, he studied medicine, chemistry and veterinary medicine in Tübingen, Munich, Würzburg and Hannover. After a short period as an assistant doctor in Würzburg from 1924 to 1926, he began as an assistant at the pharmacological institute in Halle under M. Kochmann. He qualified as a university lecturer there in 1928. He had been a senior physician in the reserve since 1927 (BArch R 4901/25,448). In 1930, he was appointed to the chair of clinical pharmacology in Hamburg. There he joined the NSDAP in 1931 (BArch R 9361-IX KARTEI/41,030,619) and co-founded the National Socialist group of lecturers at the University of Hamburg in 1932 (BArch R 4901/25,448). When W. Griesbach, the provisional head of the Institute of Pharmacology, was dismissed in 1933 due to his “non-Aryan” descent, Seel took over his position on a provisional basis until Eduard Keeser was appointed shortly afterwards. Seel was a member of the NSDÄB, NSLB, NSFK, and NSV (BArch R 4901/25,448). At the end of 1933, he went to Berlin and became head of the pharmacological department of the Kaiser Wilhelm Institute for Physical Chemistry. From 1935, he headed a “Research Center for Clinical Pharmacology” in Berlin (Catalogus Professorum Halensis 2024a). He was employed by the armed forces in 1938 during the Sudeten crisis (BArch R 4901/25,448). In 1942, Seel was appointed director of the Institute for Cancer Research in Posen. After the Allies’denazification process, Seel became a lecturer at Humboldt University in Berlin in 1947 and remained there until his retirement in 1952. Hans Seel died in Berlin on May 3, 1961 (Catalogus Professorum Halensis 2024a)
**Karl Zipf (1895–1990)**
Born on January 1, 1895 in Oberkirch (Schwarzwald), K. Zipf studied medicine in Strasbourg, Würzburg and Heidelberg and fought in the First World War. In 1919, as a soldier, he was involved in the violent end of the Würzburg Soviet Republic (Würzburger Räterepublik) (BArch R 4901/13,281). In 1924, he began as an assistant at the Institute of Pharmacology in Heidelberg and a year later moved to the Institute in Münster under H. Freund. There he habilitated in 1926 and became the first assistant. In 1933, he joined the NSDAP (BArch R 9361-IX KARTEI/50,590,109) and the SS (BArch R 9361-III/565,539), and provisionally took over the management of Freund’s institute, who, as a Jew, was expelled from his position. He was also a member of the NSDÄB and DVSTB (BArch R 4901/13,281). In 1935, Zipf was appointed to the chair in Königsberg. This had become vacant because its holder, F. Eichholtz, and his first assistant W. Keil had been appointed to Heidelberg. In Königsberg, Zipf rose through the ranks of the SS to become SS-Hauptsturmführer (BArch R 4901/13,281). For the Wehrmacht, he worked as a consultant pharmacologist (rank: Oberstabsarzt der Reserve) in the reserve army in military district 1. He is also said to have done scientific work for the Wehrmacht (ArchMHH, Dep. 13, Band 124), but nothing is known about this. After a period of time working at the German Drug Testing Institute in Munich, Zipf was appointed head of the Institute of Pharmacology at the Veterinary Faculty of the University of Munich in 1954. He retired in 1963, but continued to run the institute on a provisional basis until 1970. Karl Zipf died on March 22, 1990 in Leutershausen (Lindner and Lüllmann 1996)

The many politically motivated changes around 1945 follow the victory over National Socialism, when the Allies began to dismiss supporters of the Nazi system from their posts and fill them with persons who were considered to have moral and political integrity (Hayse 2003; Oehler-Klein and Roelcke 2007). This led to 19 (61%) of the 31 heads of university pharmacology institutes who were still in office in 1945 being removed from their posts. In the following years, 13 of them were reappointed as institute directors, mostly at the same university as before. A good example of this is the Hamburg pharmacologist Eduard Keeser, who, as Rector of the University of Hamburg from 1941 to 1945, implemented the National Socialist ideology and order at the entire university. He was removed from office by the Allies in 1945 but was allowed to return after a short time and remained the head of the university pharmacological institute in Hamburg until 1956 (Guhl 2019). This ineffectiveness of the denazification processes among the heads of pharmacological institutes is not a special case, but a good example of structures in the entire German society during this period (Hayse 2003). The slow return of Jewish scientists to German universities after 1945, as shown in this work, is a well-known phenomenon that affects many universities and disciplines (Oehler-Klein and Roelcke 2007). Politically motivated changes by National Socialism from 1933 and the changes by the Allies around 1945 were well-documented processes, which may mean that they are disproportionately represented in this work. Politically motivated changes at other times probably occurred more frequently than is documented here. This would have to be investigated on a case-by-case basis, which could not be researched or realized within the scope of this work. In summary, from 1933 onwards, many pharmacologists were expelled by the Nazis for racist reasons. Their positions were often taken over by pharmacologists who were loyal supporters of National Socialism and shared and spread its ideology. In 1945, the Allies’ attempt to permanently expel these institute directors from their posts failed because many were able to continue working a short time later.

### Political and racial motives for personnel changes

The average age of the institute directors at the time of their appointment in the period from 1933 to 1945 does not differ significantly from the average age in the years before (1918 to 1932). Therefore, there is no evidence that the National Socialism appointed significantly younger and thus presumably also less experienced pharmacologists as institute directors.

The fact that the institute directors after 1945 were on average significantly older when they took office than in the decades before is because, as already described above, the institute directors who were in office before 1945 and were partially dismissed by the Allies were reappointed to new offices shortly afterward. In summary, the average age of institute directors at the time of taking office remained the same over the years. It was shown that after 1945, it were not young new pharmacologists who were appointed as institute directors, but rather that the old pharmacologists were reappointed.

The data clearly shows the extent to which the institute directors considered as “non-Aryan” were driven out of their positions after 1933 by political and social pressures. The Nazis persecuted people not because of their beliefs, but because of their by national socialist ideology attributed “race.” Thus, not only institute directors who identify themselves as Jewish were expelled, but also institute directors who belonged to other religions like Protestantism but whose parents or grandparents were Jewish. Examples of this are Otto Riesser, Rudolf Gottlieb, and Martin Kochmann (Table 4). From 1945 to 1963, there was only one institute director (Albert Wollenberger in Berlin) who remigrated from the USA because of the political repression of left-wing people (McCarthyism) and three institute director with Jewish ancestors (Otto Riesser in Frankfurt, Franz Theodor von Brücke in Wien (Vienna) and Werner Grab in Gießen) in German-speaking countries. This can be explained by the fact that the expelled institute directors were either killed or had been given new jobs abroad.

The analyses should be viewed with caution due to the sometimes large gaps in the data. To sum up, all persons who did not fit into the Nazis’ Aryan image were also removed from the pharmacological research community.

### Political commitment and individual charge

Until 1945, around 49% of the entire medical profession were members of the NSDAP (Kater 2000). Recent studies suggest that this figure is even higher (Rüther 2001). The 63% proven NSDAP memberships of pharmacological institute directors under National Socialism are higher in direct comparison. Because no information about NSDAP membership could be found for over a third (37%) of the institute directors, the number of actual NSDAP memberships is likely to be higher. This is because, at the end of war, many NSDAP files were destroyed on purpose. A list of all institute directors by name who were members of the NSDAP, SS, and/or SA is shown in Table 3 and Fig. 10.

To compare the proportion of institute directors with confirmed NSDAP membership in terms other than number, the years of service of the institute directors between 1933 and 1945 were analyzed. This analysis also shows that the largest proportion (55%) of years of service during this period was performed by institute directors with NSDAP membership. They therefore presumably had a major influence on university research and teaching during this period.

Konrad Schübel in Erlangen and Wolfgang Heubner in Berlin were institute directors who demonstrably did not belong to the NSDAP and remained in their positions throughout the period between 1933 and 1945. There is a whole chapter in the book by Sabine Schleiermacher and Udo Schagen (2008) on W. Heubner and his role in National Socialism. Both are mentioned in Table 5. It is noticeable that both were proven combat gas experts and had already been in office for several years before 1933. Although they did not join the NSDAP, they were members of other Nazi organizations and therefore supporting National Socialism while being of great use for National Socialism. Therefore, they were not removed from office.

Looking at when the institute directors applied to join the NSDAP and when they were accepted, it can be seen that two of the institute directors (Konrad Pohle 1930 in Halle and Hans Seel 1931 in Hamburg) had already become members of the NSDAP before the National Socialists seized power in 1933, which later was very advantageous because it was considered as a special commitment to the Nazi movement. There was no political pressure becoming a member of the NSDAP at this time. Therefore, it can be assumed that these institute directors were politically convinced by the ideology of the National Socialists.

After 1933, many university lecturers applied for membership of the party due to better career prospects (Gebhardt and Grüttner 2014). Nevertheless, they knowingly and willingly accepted that they were becoming an active part of the extremist and racist National Socialism. This also explains the sharp increase in memberships around 1933. The fact that no institute directors joined the NSDAP between 1934 and 1937 was because the NSDAP imposed an admission ban for this period in order to prevent too many free riders from joining (Süß 2017). The large proportion of new memberships around 1937 can therefore be explained by the backlog from the previous years. Between 1942 and 1945, no institute directors joined the NSDAP either. For most of the NSDAP memberships considered in this study, the dates of application are missing. The dates of joining the party must also be questioned in some cases, as the NSDAP dated party memberships earlier or later for organizational reasons (Falter 2020).

A similar picture emerges with regard to the memberships of institute directors in so-called auxiliary organizations of the NSDAP, like SS and SA. Membership of these organizations was mostly voluntary, except for the NSDB. Assistants, private lecturers, and non-civil servant extraordinary professors were obliged to be members of the NSDB from 1933 onwards. Professors who had previously been civil servants were not affected. This measure served to harmonize the next generation of academics in line with National Socialism (Schilling 2003; Grüttner 2024). There is no evidence of NSDB membership for 16 pharmacologists who should have been affected by this regulation (W. Blume, A. Forst, H. Gebhardt, E. Goetze, H. Haas, F. Hauschild, H. Hofmann, M. Kiese, W. Koll, H. Konzett, L. Lendle, K. Pohle, W. Schmid, K. Soehring, R. Weigmann, and H. Zipf).

The institute directors who were active during National Socialism were, on average, more often members of such organizations than members of the German medical profession (Kater 2000). The only organization considered in which a below-average number of institute directors were members was the HJ. This can be explained by the average age of the institute directors of around 41 years when they took office during this period. If the institute directors were only appointed to office in 1945, they were on average 29 years old in 1933 and therefore already too old for the HJ (Benz et al. 1998). In summary, a large proportion of the heads of university pharmacological institutes were members of the Nazi Party or other National Socialist organizations in National Socialism and were thus an active part of this system.

### Military service and research in the second world war

A total of 29 consultant pharmacologists worked for the army (ArchMHH, Dep. 13, Band 124), their main task being “to advise the military district doctors on drug poisoning and all questions relating to combat agents” [Transl. by the author; “in der Beratung der Wehrkreisärzte bei Arzneivergiftungen und allen Fragen aus dem Bereich der Kampfstoffe”] (Neumann 2005). Ordinaries or habilitated senior physicians were appointed as advisory physicians for the various specialties (Neumann 2002). Accordingly, of the 29 consulting pharmacologists in the army, 15 were heads of university pharmacological institutes (ArchMHH, Dep. 13, Band 124).

Regarding pharmacological research for the German military, it is known that 20 of the 48 institute directors conducted such research in the Nazi era. The pharmacological institutes at the universities of Marburg (director: Hans Gremels), Danzig (director: Werner Koll), Münster (director: Ludwig Lendle), Leipzig (also under Ludwig Lendle), and Giessen (director: Fritz Hildebrandt) were involved in nerve gas research as branch offices of the Military Medical Academy (*Militärärztliche Akademie*) and the Army Ordnance Office (*Heereswaffenamt*). This research was allegedly largely concerned with protective measures against combat gases (Schmaltz 2017) and therefore of a great use for National Socialism. The Institute of the Military Medical Academy Berlin (Militärärztliche Akademie Berlin) under Professor Wirth, which partly carried out, managed, and coordinated this research, stood out particularly in this regard (Neumann 2005). As this institute was not a university, it was not considered further in this study. Other pharmacological research topics for the German military included Pervitin, a stimulant for soldiers (ArchMHH, Dep. 13, Band 141) intravenous fluid substitution (BArch R 9361-II/1,026,697) and malaria therapy (Karzel 2017a,2017b). Human experiments on pharmacological topics such as chemical warfare agents were carried out in concentration camps. This was mostly done by industry (such as I.G. Farben) on behalf of the SS. There are indications that the pharmacological department of the Military Medical Academy in Berlin, under the direction of W. Wirth, also knew about these experiments and was involved in them (Kopke and Schultz 2006). Some of the heads of the pharmacological institutes at the universities also worked for this department, and their research topics (Tabun, malaria) (Schmaltz 2006) were suited to the experiments being carried out, so that at least complicity is suspected. There are no direct sources that they were involved in human experiments in concentration camps, because the source material for this is very poor (Neumann 2005). Only W. Heubner was accused in the Nuremberg Doctors’ Trials in 1947 of knowing about the human experiments to make saltwater drinkable in the Dachau concentration camp. He denied this (Herken 1999). Summed up there is evidence that there were pharmacologists from universities researching on topics that were highly relevant for National Socialism.

## Conclusions

In a historical context, the results of this work are conclusive: The directors of university pharmacology institutes in the Nazi era were exclusively “aryan.” The results show that this group also submitted to the extremist und racist rules and ideals of National Socialism. This becomes clear from the expulsions of “non-aryan” professors from 1933 onwards and the above-average share of institute directors with memberships in the NSDAP, the SS, the SA, and other party-affiliated associations. In addition, many of the pharmacological institute directors worked for the German military. Both, in an advisory capacity in the field and conducting research in their laboratories. The extent to which the German pharmacologists at the universities influenced the war effort cannot be assessed based on the available sources.

The results also show that the Allies’ denazification measures were not sufficiently effective for pharmacologists at universities after 1945. Many full professors who worked under National Socialism continued to hold high positions for many years after 1945 and had major influence on the German pharmacological community.

The history of German pharmacology during this period has not been conclusively researched and offers scope for further considerations. For example, a more detailed network analysis of the institute directors under National Socialism and in the years after that could shed a different light on the results of this work or corroborate them. In addition, detailed analyses of individuals and their role in the Nazi era could help to reappraise the history of German-speaking pharmacology. Many sources for such research endeavors are still available in the archives of universities, federal states, and scientific societies.

## Data Availability

All source data for this work (or generated in this study) are available upon reasonable request.

## Abbreviations

BDM: Bund Deutscher Mädel (League of German Girls)
DGPT: Deutsche Gesellschaft für experimentelle und klinische Pharmakologie und Toxikologie e.V. (German Society for Experimental and Clinical Pharmacology and Toxicology)
DDR: Deutsche Demokratische Republik (German Democratic Republic)
HJ: Hitler-Jugend (Hitler Youth)
NSDÄB: Nationalsozialistischer Deutscher Ärztebund (National Socialist German Physicians’ Association)
NSAP: Naunyn-Schmiedeberg’s Archives of Pharmacology
NSDAP: Nationalsozialistische Deutsche Arbeiterpartei (National Socialist German Workers’ Party)
NSDB: Nationalsozialistischer Dozentenbund (National Socialist Teachers’ Association)
NSFK: Nationalsozialistischer Frontkämpferbund (National Socialist Front Fighters’ Association)
NSLB: Nationalsozialistischer Lehrerbund (National Socialist Teachers’ Association)
SA: Sturmabteilung (Storm Division)
SS: Schutzstaffel (Protection Squadron)
UKE: Uniklinikum Hamburg-Eppendorf
WHO: World Health Organisation
