# Correction: Historical analysis of the directors of university institutes of pharmacology in Germany and Austria in the period 1918–1963

**DOI:** 10.1007/s00210-025-04771-y

**Published:** 2025-11-01

**Authors:** Hannes Schneider, Christine Wolters, Roland Seifert

**Affiliations:** 1 Institute of Pharmacology, Hannover Medical School, Carl-Neuberg-Straße 1, 30625 Hannover, Germany; 2 Institute for Ethics, History and Philosophy of Medicine, Hannover Medical School, Carl-Neuberg-Straße 1, 30625 Hannover, Germany

**Correction: Naunyn-Schmiedeberg's Archives of Pharmacology**

10.1007/s00210-025-04342-1

In the original publication: Schneider H, Wolters C, Seifert R. Historical analysis of the directors of university institutes of pharmacology in Germany and Austria in the period 1918-1963. Naunyn Schmiedebergs Arch Pharmacol. 27 June 2025. doi: 10.1007/s00210-025-04342-1. Epub ahead of print. PMID: 40576765. A mistake was made in Table 5 regarding Fritz Külz (1887-1949). He was a member of the NSDB in Frankfurt (ISG FFM S4c No. 305; UAF Dept. 4 No. 1429; UAF Dept. 14 No. 392) and therefore a member of a Nazi organisation. There was also a typing mistake in Table 5 concerning Hans Seel (1898-1961): Seel was not a member of the NSÄ but of the NSDÄB. We apologise for any confusion this may have caused.

The original article has been corrected.

